# Latent profiles of youth social support: a study on variations and their impact on self-esteem

**DOI:** 10.3389/fpsyg.2025.1538464

**Published:** 2025-06-23

**Authors:** Jingxian Yu, Yuqin Guo, Yongqi Liang, Huan Peng, Na Li, Weisheng Gu, Hanjiao Liu

**Affiliations:** ^1^The Seventh Clinical College of Guangzhou University of Chinese Medicine, Shenzhen, China; ^2^Shenzhen Hospital of Integrated Traditional Chinese and Western Medicine, Shenzhen, China; ^3^Fujian University of Traditional Chinese Medicine, Fujian, China

**Keywords:** social support, self-esteem, young people, mental health, latent profiling

## Abstract

**Introduction:**

The current significant suicide rate reflects the urgency of addressing mental health problems among young people. At the same time, social support and self-esteem are key factors affecting young people’s mental health and suicide risk. Therefore, this study aims to explore the variations in perceived social support among youth using a latent profile analysis approach and examine its association with self-esteem.

**Methods:**

Questionnaires were distributed using a simple random sampling technique in Shenzhen and Shaoguan, Guangdong Province. Data were collected using the multidimensional perceived social support scale and the self-esteem scale, and descriptive analysis and potential profile analysis were performed using SPSS and R.

**Results:**

This study identified three potential categories of perceived social support: “High Social Support” (55.7%), “High Friend Support and Moderate Social Support” (34.35%), and “Low Social Support” (9.95%), and young people who work in the service industry, are widowed, have two or more children, and have high academic achievement are likely to have worse perceived social support. Self-esteem was positively related to the categories of perceived social support, and the group with low social support had the lowest self-esteem.

**Discussion:**

Most young people have a high level of perceived social support, but a low perceived social support group needs more attention and help. It is suggested that both social support and self-esteem should be paid attention to maintain young people’s mental health.

## 1 Introduction

With more than 720,000 deaths a year, suicide has become the third leading cause of death among young people, posing a global public health challenge (Hughes et al., 2023). Given the prominence of emotional disorders and mental distress in suicide attempts and premature deaths resulting from suicide, mental health problems among young people urgently need to be addressed and addressed to reduce the risk of suicide (Casas-Muñoz et al., 2024; Lawrence et al., 2021). Studies have shown that social support influences youth’s willingness to seek both informal and professional help, while also reducing reliance on extreme self-reliance, which is considered a barrier to accessing mental health support (Ishikawa et al., 2023). At the same time, young people, those who are overly concerned with social standards and expectations, can be hard on themselves, see their shortcomings, and feel worthless, resulting in low self-esteem, this exacerbates the fear of being socially marginalized and triggers suicidal thoughts and behaviors (Blader, 2018). Therefore, exploring young people’s social support and self-esteem can help identify their risk of mental health crises and target interventions.

Social support refers to the emotional or tangible services provided by communities, social networks, trusted family members, and partners. It primarily includes tangible support, such as receiving material assistance or direct services from others, and perceived support, which reflects an individual’s emotional experience of respect, understanding, and support (Cullen, 1994). Adequate social support enhances resilience to stress, which reduces the risk of psychological disorders, while unstoppable anxiety may occur when an individual’s stress buffer is challenged by a lack of social support (Grey et al., 2020; Sun et al., 2024). A recent meta-analysis indicated that higher levels of perceived social support and self-related traits such as self-esteem and self-efficacy were significantly associated with lower risks of depression and anxiety among adolescents and young adults (Yeo et al., 2023). Restrepo et al.’s survey of 350 college students also found that comprehending social support as a protective factor can help reduce suicidal behavior in those who are victims of interpersonal trauma (Restrepo and Spokas, 2023). Thus, understanding the level of social support among young people, sensitively identifying those who lack it, and targeting improvement measures can promote mental health and enhance their happiness and well-being.

Self-esteem is the degree to which a person cherishes, values, recognizes, or likes himself. It comes from an individual’s evaluation of himself, this includes the way you see yourself, respect for yourself, and appreciation for your value in a particular area (Doré, 2017). Self-esteem consists of two parts: implicit self-esteem, which is defined as a relatively automatic, pre-conscious, emotion-related self-assessment, and explicit self-esteem, which is rooted in rational, conscious self-assessment that may be influenced by social expectations (Buhrmester et al., 2011). Several studies have provided strong evidence for a relationship between self-esteem and mental health. A high level of self-esteem is a positive assessment of one’s overall self and a cornerstone of mental health, while a low level of self-esteem can lead to negative outcomes such as mental illness due to persistent negative self-perceptions (Cai et al., 2024). A study of young people found that self-esteem, self-compassion, self-awareness, self-efficacy, and self-regulation were negative predictors of anxiety levels, and low self-esteem leads to higher depression (Yeo et al., 2023). Recent longitudinal studies have further demonstrated a reciprocal association between self-esteem and perceived social support among adolescents, suggesting that both constructs may reinforce one another over time (Marshall et al., 2014). This reciprocal link is particularly relevant for vulnerable youth populations, where social support from peers, caregivers, or institutional staff has been found to play a crucial role in fostering adolescents’ self-worth and emotional well-being (Singstad et al., 2021). In addition, self-esteem is thought to influence self-reflection and the formation of positive perceptions of oneself, which can help young people cope with life challenges and stressors and manage emotional distress (Yeo et al., 2023).

Sociometrics theory suggests that self-esteem is part of a psychological system that stems from monitoring the interpersonal value of the self and is closely related to social support (Leary, 2005). Don et al.’s study found that individuals with low self-esteem may attempt to protect themselves from social exclusion by indirectly seeking support from their intimate partners; however, these behaviors may trigger negative partner support, further exacerbating their deficiency (Don et al., 2019). In contrast, according to Poudel et al.’s observations of adolescents, higher levels of perceived social support positively predicted their self-esteem and self-evaluation, and self-esteem played a key role in social support and mental health (Poudel et al., 2020). Beyond this, there appears to be a more complex relationship between social support and self-esteem. After observing 961 adolescents over time, Marshall et al. found that self-esteem, which refers to evaluations of oneself, predicted individuals’ perceptions of the quality of social support and the size of their support networks (Marshall et al., 2014).

The present study is grounded in the Sociometer Theory, which posits that self-esteem functions as a psychological monitor of one’s social belonging and relational value (Leary, 2005). This framework provides a useful basis for understanding how perceived social support—particularly the sense of being valued and cared for by others—may influence and be influenced by self-esteem. In addition, prior longitudinal and cross-sectional studies have established a reciprocal association between these two constructs among youth, supporting the rationale for examining them together using a person-centered approach (Marshall et al., 2014; Poudel et al., 2020).

Previously, most research on perceptions of social support has focused on testing the psychometric properties of questionnaires and using variable-centered methods (e.g., correlation analyses, and regression analyses; Lin et al., 2022; Steine et al., 2020; He et al., 2023). These analyses take full account of the relationships between variables and assume that subjects share the same characteristics, ignoring the unique patterns that may exist across the different dimensions of their perceptions of social support (Chouhy, 2019). In contrast, individual-centered approaches can identify potential population subgroups based on characteristics observed from multiple dimensions. Latent profile analysis (LPA) is one such person-centered research method that can explore population heterogeneity by clustering data with continuous explicit variables (Nylund et al., 2007). Previously, LPA has been useful in identifying characteristics of perceived social support in specific populations. A study by Mai et al. revealed that during covid-19, four types of social support existed among students aged 15–25 years old, namely, “Extremely Low Perceived Social Support Groups (ELPSSG),” “Low Perceived Social Support Group (LPSSG), Medium Perceived Social Support Group (MPSSG) and High Perceived Social Support Group (HPSSG; Mai et al., 2021). At the same time, Bai also identified four social support profiles among the 1,286 parents, namely “low,” “medium,” “high” and “Divergent” (Bai et al., 2023). Their study revealed the possibility of the existence of diverse categorizations of comprehension social support in groups, and therefore, we hypothesized 1: There are different profiles of comprehension social support in youth groups. And, given the close correlation between perceived social support and self-esteem, we propose Hypothesis 2: Young people’s level of self-esteem has a reciprocal positive correlation with the configuration of the profile of comprehending social support.

## 2 Methods

### 2.1 Survey method

From November 2023 to May 2024, this project used a simple sampling technique to randomly distribute questionnaires in Shenzhen and Shaoguan cities in Guangdong Province, taking into account the effects of heterogeneity in terms of the level of economic development and public infrastructure on the characteristics of the residents and their comprehension of social support. We chose to distribute the paper questionnaires at primary health facilities and community activity centers, where a large number of residents can often be reached. And, an online electronic questionnaire was created for the convenience of web users who wished to participate and provide their input and feedback. The study officially began after the reporter read and signed the paper or electronic informed consent form found on the front page of the questionnaire.

### 2.2 Subjects

The inclusion criteria for participants were age 18 to 35 years and informed consent with a commitment to fully comply with the study program.

Exclusion criteria included: inability to cooperate with research tasks (e.g., intellectual disabilities or abnormal cognition), confirmed diagnosis of a mental disorder; participants requiring higher levels of social support (e.g., due to disability, malignancy, or kidney failure) because of limited self-care ability or severe illness or vital organ dysfunction; and individuals with dyslexia or inability to comprehend the questionnaire content.

### 2.3 Research tools

#### 2.3.1 General information questionnaire

Socio-demographic data were collected through self-designed general information questionnaires, which included gender, age, race, education, occupation, income level, marital status, birth status, and health status.

#### 2.3.2 Perceived social support scale

The Multidimensional Perceived Social Support Scale (MSPSS) was used in this study to assess the social support of the reporter with this instrument. The instrument was designed by Zimet et al. (Dahlem et al., 1991). Jiang completed the translation and cultural adaptation of the MSPSS (Jiang, 1999). This scale has 12 entries divided into three dimensions, namely family support (items: 3, 4, 8, 11), friend support (items: 6, 7, 9, 12), and other people’s support (items: 1, 2, 5, 10) and each of these dimensions contains four items: practical help, emotional support, availability to discuss problems, and decision-making help. The instrument is scored on a 7-point Likert scale, with answers 1 through 7 indicating a range from (completely disagree) to (completely agree). The final score ranges from 12 to 84, and subscale scores can be calculated by summarizing the relevant responses. In this study, the Cronbach’s alpha (*α*) for this tool was 0.905.

#### 2.3.3 Self-esteem scale

The Self-Esteem Scale (SES), a self-report assessment tool that reflects an individual’s overall evaluation of his or her worth, was designed by Rosenberg in 1965 (Rosenberg, 1965). Ji and Yu completed the translation and assessment of the reliability and validity of the scale in China (Ji and Yu, 1999). The scale consists of five positively expressed and five negatively expressed items. The scale was hypothesized to be a one-dimensional measurement instrument based on the recommendations of Zeigler-Hill and Shackelford (2020). Although Rosenberg supported that positive scoring be used for items 1, 2, 4, 6, and 7; and that reverse scoring be used for items 3, 5, 8, 9, and 10. However, Tian suggested that adhering to the positive scoring of item 8 in the Chinese context would help to ensure the accuracy of the findings and conclusions of the study and make the whole scale highly reliable (Tian, 2006). This instrument was scored using a 4-point Likert scale (1 = Strongly Disagree; 4 = Strongly Agree) with a total score of 10–40, with higher scores being indicative of high levels of self-esteem. In this study, the Cronbach’s alpha (*α*) for this instrument was 0.790.

### 2.4 Statistical analysis

First, both researchers independently transcribed the responses into an Excel file and eliminated duplicates, incomplete information, and foreigners’ responses, which were then imported into SPSS any questions encountered during the screening process of the questionnaires were resolved by reviewing the raw data and through consultation between the two researchers. In the second step, descriptive analysis of the data (including: mean, standard deviation, median, minimum, maximum, and percentage), common method bias test, and internal consistency of the psychometric instruments were completed using SPSS 27.0. In the third step, a latent profile analysis (LPA) was completed using the estimate profiles function of the tidy LPA R package and the mclust BIC function of the mclust R package of the R software version 4.4.1 to identify the optimal number of latent profiles for the youth group’s perceptions of each dimension of social support (Rosenberg et al., 2018; Fraley et al., 2012). We tested models with 1 to 5 profiles. Several fit indices helped determine the best class solution, including the Akaike Information Criterion (AIC) and Bayesian Information Criterion (BIC), when a model possessed lower AIC and BIC indicating superior fit (Tofighi and Enders, 2008); Entropy, used to assess the confidence that a participant was categorized as belonging to one profile or the other, with the closer it was to 1 indicating that the model possessed a higher level of classification accuracy (Lubke and Muthén, 2007); Bootstrap-Likelihood-Ratio-Test (BLRT), which possesses the trait of *p* < 0.05 can help determine the superiority of the K-class model over the K-1-class model (Sinha et al., 2021); and Expectation Maximization (EM), where obtaining a larger value indicates a high level of classification accuracy (Fraley and Raftery, 1998). In addition to this, the number of participants assigned to each group (we accept that the smallest configuration is >5% of the overall) and the simplicity, interpretability, and coherence of the theory are also important reference information for determining the final number of subgroups of comprehension social support (Tein et al., 2013). In the fourth step, with reference to the characteristics of the configurations obtained above, descriptive analyses and multivariate logistic regressions were considered for estimating the predictive role of socio-demographic variables on the shift in the configuration of the PSSS. Finally, one-way ANOVA and *post-hoc* tests helped to assess the relationship between the configuration of a given PSSS and self-esteem. In this project, two-sided tests were used for all statistical tests, and *p* < 0.05 was considered statistically significant.

### 2.5 Ethics declaration

Before the official start of the study, we obtained ethical review and approval from the Review Committee of the Seventh Clinical Medical College of Guangzhou University of Traditional Chinese Medicine (Approval No. KY-2024-026-01). All project team members strictly adhered to the Helsinki Declaration and its amendments throughout the study (Bierer, 2025). Participants were informed about the purpose and procedures of the study, they participated voluntarily and there were no foreseeable risks or harm in this study. The study started after the participants read and signed a consistent informed consent form, and they had the right to withdraw freely during the study. Any questions related to this project were answered by the participants from the researcher either face-to-face or by e-mail. All the data were transcribed by two researchers and proofread in a password-protected Excel file. The paper questionnaires were stored in an opaque sealed document bag while the electronic questionnaires were kept in a password-protected electronic folder.

## 3 Results

### 3.1 Characteristics of participants

A total of 704 questionnaires were recovered in this survey, but we excluded 81 of them after team consultation due to a variety of reasons, they include duplicates (*n* = 38), incomplete information (*n* = 3), age discrepancy (*n* = 17) and completion time <120 s (*n* = 23), which was personally experimented with by the investigator and was deemed to be insufficient to support the completion of the questionnaire content. Finally, 623 valid questionnaires were collected, with a validity rate of 88.5%. Participants included 429 females (68.9%), 90% were tertiary educated (561), more than half were in the workforce (53%), 51.4% (320) were in a relationship or married, 56.2% (350) had not yet given birth and a total of 341 (54.7%) self-reported being in a healthy state. More detailed information is available in Table 1.

**Table 1:** General information of the participants.

Characteristics	Total (*N* = 623)
Gender (n,%)	
Male	194 (31.1%)
Female	429 (68.9%)
Age (n,%)	
18–23	341 (54.7%)
24–29	234 (37.6%)
30–35	48 (7.7%)
Nation (n,%)	
Han Nationality	569 (95.7%)
Minority Nationality	27 (4.3%)
Education Attainment (n,%)	
Secondary school	2 (0.3%)
Junior High School	10 (1.6%)
High School or Secondary Vocational School	30 (4.8%)
Junior College or University	471 (75.6%)
Postgraduate Student	110 (17.7%)
Career (n,%)	
Students	293 (47%)
Service Sector	66 (10.6%)
Manufacturing Industry	37 (5.9%)
Office Clerk	116 (18.6%)
High-Tech Industry	87 (14%)
Unemployed	24 (3.9%)
Salary Level (n,%)	
Not yet employed, and no wages	297 (47.7%)
Less than 50,000 yuan/year	63 (10.1%)
50,000–100,000 yuan/year	167 (26.8%)
100–200,000 yuan/year	78 (12.5%)
More than 200,000 yuan/year	18 (2.9%)
Marital Status (n,%)	
Single	290 (46.5%)
In a Relationship	252 (40.4%)
Married	68 (10.9%)
Divorced	12 (1.9%)
widowhood	1 (0.2%)
Fertility Status (n,%)	
Childless	350 (56.2%)
Have A Child	202 (32.4%)
Have Two Children	52 (8.3%)
Three Or More Children	19 (3.0%)
Health Status (n,%)	
Healthy	341 (54.7%)
Sub-Healthy	206 (33.1%)
Chronic Non-Communicable Diseases	60 (9.6%)
Rehabilitation Period For Acute Illnesses	16 (2.6%)

### 3.2 Common method bias test

Since all data are collected through a single method, primarily self-reporting, this may introduce a common method bias. Therefore, we use the Harman single-factor method to test this deviation (Harman, 1960). Referring to Hair’s recommendations, the largest factor accounted for < 40% of the variance explanation, indicating that the data had an acceptable common method bias (Hair et al., 1998). Finally, we identified five factors with eigenvalues greater than 1, and the unrotated first factor explained only 35.5%. Therefore, it can be concluded that there is no significant common method bias in this study.

### 3.3 The underlying profile and characteristics of perceived social support

The results of the LPA are shown in Table 2. Comparing the results of the model analysis for configurations 1–5, configuration 5 demonstrated the lowest AIC and BIC, which seemed to be the most suitable solution. Moreover, according to the BLRT criteria, the 5-profile model was also more suitable than the previous model (*p* < 0.05). However, concerning the results of the mclust clustering analysis, configuration 3, which possessed the highest value of mclustBIC, was considered the most recommended model (see Table 3 for details). In the end, after carefully comparing the AIC and BIC, BLRT, entropy, and theoretical interpretability of each configuration, we chose the 3-profile model. It has acceptable goodness-of-fit with AIC of 10701.69 and BIC of 10790.39; classification accuracy with BLRT < 0.01; and classification precision with entropy of 0.83.

**Table 2:** Fitting index of latent profile analysis about the perceived social support of youth groups.

No. of profiles	AIC	BIC	Entropy	BLRT	n(%) per profile
1	11320.63	11347.24	–	–	623 (100%)
2	10890.2	10947.85	0.69	0.01	338 (54.25%)/285 (45.75%)
3	10701.69	10790.39	0.83	0.01	347 (55.70%)/214 (34.35%)/62 (9.95%)
4	10625.39	10745.12	0.76	0.01	224 (35.95%)/236 (37.88%)/89 (14.29%)/74 (11.88%)
5	10568.39	10719.17	0.77	0.01	134 (21.51%)/113 (18.14%)/253 (40.61%)/47 (7.54%)/76 (12.20%)

AIC, Akaike information criterion; BIC, Bayesian information criterion; BLRT, bootstrap likelihood ratio test.

**Table 3:** Classification accuracy of latent profiles.

Potential profile	1	2	3
1	1.000	0.000	0.000
2	0.000	0.961	0.039
3	0.006	0.019	0.974

**Figure 1:**
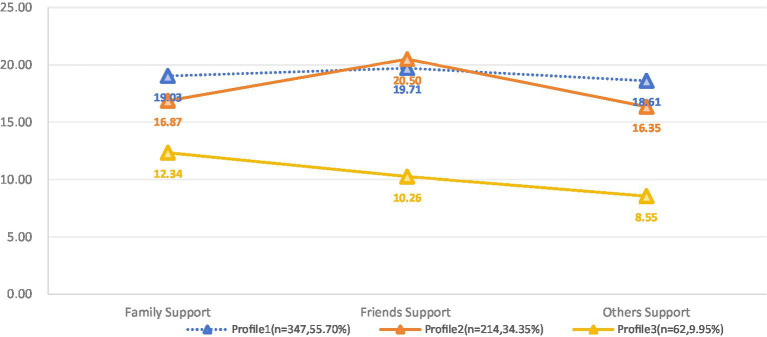
Performance of three profiles in different dimensions of perceived social support. Profile 1, High Social Support; Profile 2, High Friend Support and Medium Social Support; Profile 3, Low Social Support.

Based on the results of the latent profile analysis, we summarized and plotted the characteristics of the three profiles in line graphs, which are shown in Table 4 and Figure 1. Profile 1 consisted of 347 participants (55.7%) whose profiles showed significant strengths on all dimensions of the total score and perceived social support. Therefore, we named this profile “High Social Support.” Profile 2 consists of 214 individuals (34.35%), and although it shows moderate perceived social support in the total score, it shows a particularly high level in the “friend support” dimension. Therefore, it was named “High Friend Support and Medium Social Support.” Profile 3 was the smallest subgroup, comprising 62 participants (9.95% of the total sample). Those of the reporters assigned to this group possessed significantly lower than average total scores and showed an overwhelming weakness in all three themes of perceived social support. As a result, this profile was named “Low Social Support.”

**Table 4:** The performance of each section in understanding different dimensions of perceived social support.

Variables	Profile 1 (*n* = 347) M ± SE	Profile 2 (*n* = 214) M ± SE	Profile 3 (*n* = 62) M ± SE	F	*p*	K-W test
Family Support	19.03 ± 4.444	16.87 ± 5.442	12.34 ± 4.258	55.293	<0.001*	3 < 2 < 1
Friends Support	19.71 ± 3.846	20.50 ± 4.382	10.26 ± 2.172	176.076	<0.001*	3 < 1 < 2
Others Support	18.61 ± 3.830	16.35 ± 4.367	8.55 ± 2.215	177.502	<0.001*	3 < 2 < 1

K-W test, Kruskal-Wallis test; M, Mean; SE, Standard Error. The asterisk symbol (*) indicates statistical significance at *p* < 0.001.

### 3.4 Participant characteristics based on potential profiles

Descriptive analysis and one-way ANOVA helped us understand the sociodemographic characteristics of each profile, as shown in Table 5. Gender, education level, occupation, income level, marital status, fertility status, and health status are related to the distribution of perceived social support and possible profiles. People in the “High Social Support” group were more likely to report their health status; “High Friend Support and Moderate Social Support” status may be more common in women; People who work, are married, have two children, and are in poor health are more likely to show “Low Social Support.”

**Table 5:** Participant characteristics based on potential profiles.

Variables	Profile 1 n(%)	Profile 2 n(%)	Profile 3 n(%)	X^2^	*p*
Gender				9.097	0.011
Male	118 (34.0%)	51 (23.8%)	25 (40.3%)		
Female	229 (66.0%)	163 (76.2%)	37 (59.7%)		
Age				4.845	0.304
18–23	184 (53.0%)	115 (53.7%)	42 (67.7%)		
24–29	136 (39.2%)	82 (38.3%)	16 (25.8%)		
30–35	27 (7.8%)	17 (7.9%)	4 (6.5%)		
Nation				1.383	0.501
Han Nationality	330 (95.1%)	205 (95.8%)	61 (98.4%)		
Minority Nationality	17 (4.9%)	9 (4.2%)	1 (1.6%)		
Education Attainment				47.182	<0.001
Secondary School	1 (0.3%)	0 (0.0%)	1 (1.6%)		
Junior High School	3 (0.9%)	1 (0.5%)	6 (9.7%)		
High School or Secondary Vocational School	14 (4%)	11 (5.1%)	5 (8.1%)		
Junior College or University	272 (78.4%)	151 (70.6%)	48 (77.4%)		
Postgraduate Student	57 (16.4%)	51 (23.8%)	2 (3.2%)		
Career				41.583	<0.001
Students	171 (49.3%)	108 (50.5%)	14 (22.6%)		
Service Sector	45 (13.0%)	12 (5.6%)	9 (14.5%)		
Manufacturing Industry	20 (5.8%)	17 (7.9%)	0 (0.0%)		
Office Clerk	53 (15.3%)	38 (17.8%)	25 (40.3%)		
High-Tech Industry	46 (13.3%)	29 (13.6%)	12 (19.4%)		
Unemployed	12 (3.5%)	10 (4.7%)	2 (3.2%)		
Salary Level				29.725	<0.001
Not Yet Employed, and No Wages	178 (51.3%)	107 (50.0%)	12 (19.4%)		
Less Than 50,000 Yuan/Year	32 (9.2%)	24 (11.2%)	7 (11.3%)		
50,000–100,000 Yuan/Year	92 (26.5%)	45 (21.0%)	30 (48.4%)		
100–200,000 Yuan/Year	35 (10.1%)	32 (15.0%)	11 (17.7%)		
More Than 200,000 Yuan/Year	10 (2.9%)	6 (2.8%)	2 (3.2%)		
Marital Status				130.618	<0.001
Single	157 (45.2%)	118 (55.1%)	15 (24.2%)		
In a Relationship	160 (46.1%)	78 (36.4%)	14 (22.6%)		
Married	26 (7.5%)	10 (4.7%)	32 (51.6%)		
Divorced	3 (0.9%)	8 (3.7%)	1 (1.6%)		
widowhood	1 (0.3%)	0 (0.0%)	0 (0.0%)		
Fertility Status				66.986	<0.001
Childless	188 (54.2%)	142 (66.4%)	20 (32.3%)		
Have A Child	121 (34.9%)	59 (27.6%)	22 (35.5%)		
Have Two Children	27 (7.8%)	5 (2.3%)	20 (32.3%)		
Three Or More Children	11 (3.2%)	8 (3.7%)	0 (0.0%)		
Health Status				36.078	<0.001
Healthy	200 (57.6%)	110 (51.4%)	31 (50.0%)		
Sub-Healthy	114 (32.9%)	79 (36.9%)	13 (21.0%)		
Chronic Non-Communicable Diseases	26 (7.5%)	16 (7.5%)	18 (29.0%)		
Rehabilitation Period For Acute Illnesses	7 (2.0%)	9 (4.2%)	0 (0.0%)		

### 3.5 Potential profile membership predictors

To identify predictors of attribution for the profiles, we included the revealed demographic factors with potential in a multinomial logistic regression, using the High Social Support group as a reference.

In Profile 2, participants in the Service Sector reported lower perceived social support compared to Unemployed; those who were widowed reported worse perceived social support compared to Married, in a Relationship, and Single; and those who were childless or had only one child had higher perceived social support than those with three or more children. Graduate degrees, participants appear to exhibit poorer perceived social support than those with middle and high school degrees. See Table 6 for more information.

**Table 6:** Potential profile membership predictors.

Variables (reference items)	B	SE	OR	95%CI	*p*
Profile 2 (reference: Profile 1)					
Gender (reference: Female)					
Male	−0.228	0.214	0.796	0.523–1.210	0.285
Education Attainment (reference: Postgraduate Student)					
Secondary School	−19.867	8611.166	/	/	0.998
Junior High School	0.366	1.329	1.442	0.107–19.533	0.783
High School or Secondary Vocational School	0.330	0.494	1.392	0.529–3.664	0.503
Junior College or University	−0.320	0.240	0.726	0.454–1.162	0.182
Career (reference: Unemployed)					
Students	−0.461	0.471	0.631	0.250–1.589	0.328
Service Sector	−1.516	0.649	0.220	0.062–0.784	0.020
Manufacturing Industry	−0.927	0.678	0.396	0.105–1.496	0.172
Office Clerk	−0.727	0.583	0.483	0.154–1.513	0.212
High-Tech Industry	−0.762	0.605	0.467	0.143–1.528	0.208
Salary Level (reference: More than 200,000 yuan/year)					
Not yet employed, and no wages	−0.575	0.696	0.563	0.144–2.199	0.408
Less than 50,000 yuan/year	0.052	0.651	1.053	0.294–3.771	0.937
50,000–100,000 yuan/year	0.006	0.601	1.006	0.309–3.267	0.993
100–200,000 yuan/year	0.257	0.62	1.293	0.383–4.362	0.679
Marital Status (reference: widowhood)					
Single	18.060	1.619	69693282.450	2916353.078–1,665,488,879	<0.001
In a Relationship	18.009	1.598	66259698.230	2893234.927–1,517,452,858	<0.001
Married	18.376	1.518	95665801.380	4878728.051–1,875,887,621	<0.001
Divorced	21.474	0.000	2118085522.000		/
Fertility Status (reference: Three Or More Children)					
Childless	4.296	1.788	73.390	2.205–2442.631	0.016
Have A Child	3.842	1.772	46.629	1.446–1503.853	0.030
Have Two Children	2.462	1.757	11.730	0.375–367.038	0.161
Health Status (reference: Rehabilitation Period For Acute Illnesses)					
Healthy	−2.517	1.309	0.081	0.006–1.050	0.055
Sub-Healthy	−2.244	1.31	0.106	0.008–1.381	0.087
Chronic Non-Communicable Diseases	−1.789	1.341	0.167	0.012–2.316	0.182
Profile 3 (reference: Profile1)					
Gender (reference: Female)					
Male	0.652	0.389	1.92	0.895–4.118	0.094
Education Attainment (reference: Postgraduate Student)					
Secondary School	0.653	2.170	1.921	0.027–135.041	0.763
Junior High School	3.584	4.452	36.007	2.090–620.287	0.014
High School or Secondary Vocational School	2.122	1.022	8.346	1.127–61.802	0.038
Junior College or University	1.537	0.826	4.650	0.920–23.489	0.063
Career (reference: Unemployed)					
Students	0.437	1.018	1.547	0.211–11.374	0.668
Service Sector	−0.345	1.152	0.708	0.074–6.763	0.764
Manufacturing Industry	−16.876	1670.828	/	/	0.992
Office Clerk	−0.064	1.133	0.938	0.108–8.646	0.955
High-Tech Industry	−0.447	1.180	0.640	0.063–6.457	0.705
Salary Level (reference: More than 200,000 yuan/year)					
Not yet employed, and no wages	−0.785	1.282	0.456	0.037–5.629	0.540
Less than 50,000 yuan/year	0.843	1.210	2.324	0.217–24.892	0.486
50,000–100,000 yuan/year	0.882	1.140	2.415	0.258–22.558	0.439
100–200,000 yuan/year	0.077	1.185	1.080	0.106–11.013	0.948
Marital Status (reference: widowhood)					
Single	2.912	14884.404	18.400	/	1.000
In a Relationship	2.550	14884.404	12.806	/	1.000
Married	6.278	14884.404	532.506	/	1.000
Divorced	7.048	14884.404	1150.214	/	1.000
Fertility Status (reference: Three Or More Children)					
Childless	18.649	1906.627	125646671.000	/	0.992
Have A Child	18.448	1906.627	102787896.400	/	0.992
Have Two Children	17.276	1906.627	31825157.770	/	0.993
Health Status (reference: Rehabilitation Period For Acute Illnesses)					
Healthy	15.977	1958.287	8686350.908	/	0.993
Sub-Healthy	15.217	1958.287	4061509.873	/	0.994
Chronic Non-Communicable Diseases	16.393	1958.287	13158581.300	/	0.993

SE, standard deviation; CI, confidence interval; Due to quasi-complete separation, the related parameters were inestimable and thus denoted by “/”.

### 3.6 The relationship between perceived social support profile and self-esteem

When examining the associations between levels of perceived social support and self-esteem separately, we found that participants in the “High Friend Support and Medium Social Support” group had poorer self-esteem than participants in the “High Social Support” group. And, unsurprisingly, participants in the “low Social Support” group reported the lowest self-esteem. (Table 7).

**Table 7:** The relationship between perceived social support profile and self-esteem.

Variables	Profile 1 (*n* = 347) M ± SE	Profile 2 (*n* = 214) M ± SE	Profile 3 (*n* = 62) M ± SE	F	*p*	*Post-hoc* test
Self-Esteem	28.61 ± 4.971	27.73 ± 5.206	24.08 ± 3.335	22.412	**<**0.001	3 < 2 < 1

M, mean; SE, standard deviation.

## 4 Discussion

To the best of the authors’ knowledge, the current study is the first effort to explore potential characteristics of perceived social support in a youth population through person-centered analyses. As shown in Hypothesis 1, there are different characteristics of perceived social support among young people and the LPA results support a 3-character model labeled as follows: ‘High Social support’, ‘High Friend Support with Medium Social Support’, and “Low Social Support.” At the same time, Hypothesis 2 was verified, and there was a positive correlation between the profile of perceived social support and self-esteem.

This study successfully identified three different profiles of perceived social support among 623 individuals. Profile 1, “High Social Support,” was the largest subgroup, with 347 participants, or 55.7% of the total. Profile 2, characterized as “High Friend Support and Medium Social Support,” was the second largest group, consisting of 214 participants, or 34.35% of the total. Profile 3 is the smallest group, with 62 members, accounting for only 9.95% of the total. In comparing the profiles obtained in the studies of Mai et al. (2021) and Bai et al. (2023) with ours, we found some similarities. In both studies, the same as our findings were identified, two profiles showed overall high and low performance in comprehension of social support.

However, we failed to identify the profile characterized by very low social support, as found by Mai et al. among students in COVID administration. Moreover, compared to Mai and Bai’s study, the percentage of participants with “Low Social Support” in this program was lower, while the “High Social Support” group had a more positive response. This may be attributed to the National Health Commission of China’s (NHC) transition to a COVID-19 management strategy, which allowed young people to gradually resume offline interactions with family and friends, thereby enhancing their perception of real-life social support (Ji et al., 2022). Meshi et al. demonstrated that perceived social support from face-to-face interactions reduced the risk of problematic social media use, compared to social support obtained through social media platforms. The risk of problematic social media use is associated with reduced depression, anxiety, and social isolation, with a mental health-promoting effect (Meshi and Ellithorpe, 2021). In addition to this, we identified profile 2, characterized by high friend support and moderate social support, which seems to be a subgroup-specific to young people. As young adults, those who left their hometowns for school or job search experienced dramatic changes in their social networks, which may have affected their perceptions of social support. Lee et al. found that support from friends, but not from family, helped buffer college students from the link between perceived stress and loneliness. And, thanks to the longevity and stability of friendships, support from friends also performed better than support provided by romantic partners in buffering young people from the challenges of facing stress (Lee and Goldstein, 2016). Meanwhile, through an emotional support experiment and fMRI measurements with 71 participants, Morese et al. validated the positive effects of support from friends in reducing negative emotions (Morese et al., 2019). In summary, young people should increase face-to-face interactions with family and friends to enhance comprehension of social support and promote mental health. Moreover, the special role of friends in young people’s social support should be emphasized more, especially among those who have left home and parents.

Based on the demographic specificity of each profile, we find that young people in the service sector, widowed, with three or more children, and with graduate degrees appear to have a poorer profile of perceived social support. First, young people working in the service industry showed frail perceived social support. Because service industry workers are frequently involved in social interactions with consumers, they have to face challenges from emotional labor (Zapf and Holz, 2006). Sora et al.’s study noted that work-related emotional dysregulation may affect employees’ job satisfaction and willingness to leave their jobs, and that support from co-workers may help to regulate their negative feelings about their jobs, whereas support from the organization did not show the same effect (Sora and Vera, 2020). However, if young people and/or their coworkers lack emotional maturity and social competence, navigating social support can be limited or even trigger work-related anger interpersonal difficulties, and hostility with others, which can further exacerbate impairments in navigating social support (Fitzgerald et al., 2003). However, as only one participant in our sample identified as widowed, this finding should be interpreted with caution. The result may not be representative of the broader population of bereaved young individuals and requires further validation in future studies. Romantic partners are an important source of social support, and with the loss of a spouse and the special emotional support they provide, bereaved individuals experience serious challenges from feelings of loneliness. The emotional and social support gained through increased interactions with children, friends, and relatives does not fully buffer the bereaved from stress (Freak-Poli et al., 2022). This may explain the poorer self-assessment of comprehending social support among young people who have lost a romantic partner. Third, those with no children or only 1 child had better performance in navigating social support compared to those who had 3 or more children. Parents bear a great deal of caregiving stress during the child-rearing process. At this time, having access to a social support network of family members or peers can help increase their perception of social support and ease their emotional burden (O'Neill et al., 2019). However, when their support needs in child care exceed the affordability of their social support network, their perceived social support is thwarted and their happiness and well-being, as well as that of their children, are impacted (Geweniger et al., 2024). Even if this inadequate social support may be relative. Finally, young adults with graduate degrees appear to exhibit lower perceived social support than their peers with only a middle or high school degree. High academic achievers typically face more expectations and demands from themselves and others, which may trigger their perfectionism (Schilder et al., 2021). Dobos et al. suggest that the potential of perfectionism in reinforcing the pressures of social prescriptions on young people, as well as the perfectionist’s tendency to be socially disconnected and hostile, may influence the negative correlation between perceived social support and perfectionism (Dobos et al., 2021). This also explains the manifestation of impoverishment in comprehending social support among highly educated young people. In summary, the perceived social support status of young people who are in the service sector, widowed, have three or more children and have high academic achievement such as a graduate degree deserves more attention and active assistance.

Consistent with previous findings, there is a positive correlation between perceived social support and self-esteem (Singstad et al., 2021; Yuan et al., 2022), and we argue that there is a reciprocal relationship between the two, rather than a unilateral effect of either variable on the other. Those who excel in self-esteem are more likely to be assigned to configurations of high perceived social support. Although the ability to receive and perceive social support is innate, an individual’s appreciation of their social support is influenced by the environment in which they were raised (Taylor, 2020). Brockner suggests that self-esteem is related to an individual’s behavioral plasticity and that individuals with low self-esteem are more susceptible to their social environments than those with high self-esteem, which makes them more sensitive to the evaluations and social feedback of others and affects their social relationships (Brockner, 1988). Therefore, if young people grow up with a lack of self-confidence, it is difficult for them to form rich and close social relationships, which also determines their weakness in comprehending social support. At the same time, comprehending social support is also an important factor that affects the level of self-esteem of an individual. The sociometrics theory states that the pursuit of a positive evaluation of the value of one’s interpersonal relationships is an important source of self-esteem (Leary, 2005). Therefore, the status of social relationships and social support will influence an individual’s judgment of his or her level of self-esteem. Lu et al. explained the positive effect of perceived social support on self-esteem from the perspective of cognitive neuroscience by measuring the gray matter volume of the hippocampus and amygdala of 243 young people (Lu et al., 2023). Meanwhile, Kazi also found that social support consisting of emotional and tangible support can help improve self-esteem among married women in Riyadh, Saudi Arabia. Overall, there is a reciprocal positive relationship between perceived social support and self-esteem (Kazi, 2021). Given the impact of perceived social support and self-esteem on young people’s mental health, we believe that proactive assessment and targeted provision of measures to maintain young people’s self-esteem and perceived social support are necessary (Karaca et al., 2019).

These findings offer meaningful implications for mental health interventions targeting young populations. For instance, young adults in the service sector or those experiencing bereavement or caregiving stress may benefit from support systems that enhance their perceived relational value. Previous research has demonstrated that emotional and tangible support are significant predictors of higher self-esteem (Kazi, 2021), and that interventions enhancing self-esteem and perceived support are protective factors for mental health in young adults (Karaca et al., 2019). School-based and workplace-based mental health programs can therefore incorporate self-esteem enhancement modules alongside social support development strategies. Additionally, our findings suggest that tailored interventions should consider subgroup-specific vulnerabilities—such as social withdrawal among widowed youth or perfectionistic stress among highly educated individuals—to more effectively foster psychological resilience and well-being.

## 5 Limitation

Because we focused primarily on the relationship between perceived social support profiles and self-esteem among young people, the representativeness and generalizability of the sample were limited, so the results of this study may not apply to groups other than youth. Additionally, the number of participants in specific subgroups, such as widowed individuals, was extremely small, limiting the generalizability of those subgroup-related findings. In addition, the project uses questionnaires and lacks reports on the real experiences of youth groups in perceived social support and their views on allocation. In the follow-up work, we will further explore and improve the deficiencies.

## 6 Conclusion

Based on the reports of 623 young participants, this study identified a total of three configurations of perceived social support, which were characterized as “high Social Support,” “High Friend Support with Moderate Social Support,” and “Low Social Support.” Overall, the majority of young people exhibit moderate to high levels of perceived social support, however, Groups with low perceived social support should receive targeted interventions, as their diminished support levels may severely compromise both physical and mental health. In addition, increased attention should be paid to young people who are in the service sector, who are widowed, who have three or more children, and who have high levels of academic achievement, as they are more likely to lack social support. Finally, given the strong positive correlation between self-esteem and perceived social support, focusing on both and targeting improvement measures is the best option for safeguarding the mental health of young people.

## Data Availability

The raw data supporting the conclusions of this article will be made available by the authors, without undue reservation.
